# ECT2 promotes the occurrence and is a prognostic biomarker of head and neck squamous cell carcinoma

**DOI:** 10.7150/jca.95515

**Published:** 2024-06-03

**Authors:** Shixiong Peng, Jinhang Wang, Naiheng Hei, Zifeng Cui

**Affiliations:** 1 Department of stomatology, The Fourth Hospital of Hebei Medical University, Jiankang Road 12, Shijiazhuang, 050011, Hebei, China.; 2 Department of Stomatology, The Second Hospital of Shijiazhuang, Huaxi Road 53, Shijiazhuang, 050051, Hebei, China.

**Keywords:** Immune infiltration, Epithelial Cell Transforming Sequence 2, Head and neck squamous cell carcinoma, Prognosis, Independent prognostic factor

## Abstract

**Background:** Epithelial Cell Transforming Sequence 2 (ECT2) has been implicated in various tumorigenic processes, including proliferation, migration, and invasion. However, its specific role in head and neck squamous cell carcinoma (HNSCC) remains unclear.

**Methods:** This study integrates transcriptomic and single-cell RNA sequencing (scRNA-seq) data to explore the potential role of ECT2 in HNSCC. Differential expression analysis, cell-based assays (including CCK-8 for proliferation, transwell for migration, invasion assays, and flow cytometry for apoptosis and cell cycle analysis), and enrichment analysis were employed to investigate ECT2 expression levels and its regulatory effects on cellular phenotypes. Additionally, Mendelian randomization analysis was utilized to identify genes causally related to HNSCC using publicly available Genome-Wide Association Study (GWAS) data.

**Results:** ECT2 is highly expressed in HNSCC samples and its downregulation inhibits proliferation, migration, invasion, induces apoptosis, and affects the cell cycle transition in HSC-3 cells. Furthermore, differential analysis revealed significant differences in the immune microenvironment and drug sensitivity between high and low ECT2 expression groups. The pathways enriched in different groups include CCR and its related chemokines, as well as HLA in antigen presentation and immune response. There are also significant differences in the sensitivity to drugs such as bortezomib and dasatinib between the two groups. Prognostic models constructed from prognosis-related genes showed significant differences in prognosis between high and low-risk groups. Integration of scRNA-seq data identified Monocyte clusters as high-scoring cell clusters based on genes interacting with ECT2.Mendelian randomization analysis identified three genes (LGALS2, SLC11A1, and TKT) causally related to HNSCC within this cell cluster.

Conclusion: The findings suggest that ECT2 overexpression is associated with the survival rate of HNSCC, indicating its potential as a prognostic biomarker for this malignancy.

## Introduction

Head and neck squamous cell carcinoma (HNSCC) is a common malignant tumor that frequently occurs in the oral cavity, pharynx, larynx, and nasal cavity. According to the World Health Organization, hundreds of thousands of people worldwide are diagnosed with HNSCC annually 1, 2. Some patients miss the optimal treatment window due to late diagnosis, resulting in poor treatment outcomes. Despite some progress in recent years, the etiology of HNSCC remains incompletely understood, and treatment outcomes remain limited 3. Therefore, the identification of novel therapeutic targets and prognostic markers is of paramount importance for improving the survival rate and quality of life of HNSCC patients.

Epithelial Cell Transforming Sequence 2 (ECT2) is a gene encoding a guanine nucleotide exchange factor and transforming protein 4, 5. It plays a crucial role in the regulation of the cell cycle and maintenance of cell polarity. ECT2 is particularly important during cell division, where it orchestrates the dynamic processes of cytoskeletal reorganization and cytokinesis, thereby influencing normal cell division 6, 7. Its involvement has been implicated in the occurrence and progression of various tumors, with its overexpression linked to enhanced proliferation, migration, and invasion of tumor cells 8, 9. For instance, Mohamed A. Soltan and colleagues identified ECT2 as a potential oncogene and a prognostic and immune biomarker for various cancers 10. Chunjie Wen *et al.* found that CircSETD3 mediates acquired resistance to gefitinib in non-small cell lung cancer cells through the FXR1/ECT2 pathway 11. Additionally, researchers have found that miRNA-223-3p regulates ECT2 via the Wnt/β-catenin signaling pathway to promote proliferation, invasion, and metastasis in gastric cancer 12. The study by Sun *et al.* found that ECT2 regulates the expression of VEGF and MMP9 through the RhoA-ERK signaling pathway in patients with esophageal squamous cell carcinoma 13. Zhu *et al.* explored the correlation between the high expression of ECT2 in laryngeal cancer and its association with low differentiation, advanced stage, lymph node metastasis, and poor survival rates 14. Daizaburo Hirata *et al.* discovered through immunohistochemical staining that high expression of ECT2 is associated with poor prognosis in patients with non-small cell lung cancer and esophageal squamous cell carcinoma 15. Similarly, the results of the study by Iyoda *et al.* suggest that ECT2 serves as a proliferation marker in oral squamous cell carcinoma cells and may be a potential therapeutic target 16.

Our previous studies have shown dysregulation of ECT2 expression in HNSCC 17. However, the effects of ECT2 on various aspects of HNSCC cell phenotype, functional pathways, and immunity have not been investigated. This article first identified the expression level of ECT2 in HNSCC and its regulatory role on cell phenotypes through cell proliferation assay using cck-8, cell migration and invasion assays using transwell, and flow cytometry analysis of cell apoptosis and cell cycle, indicating the potential importance of the ECT2 in HNSCC. Subsequently, patients were classified into high and low expression groups based on the expression of the ECT2. The association between ECT2 expression subgroups and the immune microenvironment, functional pathways, and drug sensitivity in head and neck cancer was evaluated. DEGs between high and low expression groups of ECT2 were assessed. Then, LASSO-COX regression analysis was employed to identify prognostic biomarkers related to HNSCC prognosis, and predictive prognostic models and nomograms for head and neck cancer OS with medium to high accuracy were constructed. Patients were divided into high and low-risk groups based on the median value of the risk score. Using ssGSEA analysis and correlation analysis, the association between prognostic genes and immune cells in head and neck cancer was identified. Furthermore, through single-cell sequencing (scRNA-seq) data analysis, key pathways of interaction between high-scoring cell populations evaluated by prognostic genes and other cell populations were identified. Finally, based on Mendelian randomization analysis, genes causally related to HNSCC risk were identified from DEGs between high-scoring cell populations and other cell populations.

## Methods

### Data Set Acquisition

In this study, transcriptomic data of the TCGA-HNSC cohort (including 44 control samples and 522 HNSCC samples), along with corresponding overall survival (OS) survival data and clinical characteristics (Age, Gender, T stage, M stage, N stage, Grade, and Stage) for all samples were collected from the TCGA database. When conducting survival analysis, we removed samples without survival data and patients with a survival time of less than 30 days, resulting in 511 patients being used for survival analysis. Transcriptomic data of 270 HNSCC samples and corresponding OS data were obtained from the GSE65858 dataset in the GEO database. Samples from the TCGA and GEO databases were utilized as the training and testing sets, respectively. Additionally, single-cell transcriptomic (scRNA-seq) data of 63 HNSCC samples were collected from datasets in the GEO database.

For the MR analysis, genes selected through the optimal correlation analysis algorithm from the IEU database (https://gwas.mrcieu.ac.uk/) were used for expression quantitative trait loci (eQTLs). The GWAS data for HNSCC were sourced from the ieu-b-4912 dataset in the EBI database (https://www.ebi.ac.uk/gwas/).

### Differential Expression Analysis and GSEA Enrichment Analysis

Differential expression analysis between high and low ECT2 expression groups was conducted using the "limma" package in R software. A total of 232 Differentially Expressed Genes (DEGs) were retained. The selection threshold was set as an absolute value of logFC greater than 0.8 and an adjusted p-value less than 0.05. Gene Ontology (GO) enrichment analysis was performed using the "clusterProfiler" package in R software to explore pathway information associated with DEGs. Gene Set Enrichment Analysis (GSEA) was employed to investigate the mechanisms of ECT2 in HNSCC. The "GSEA" package in R software was utilized to perform enrichment analysis against the MSigDB database (https://www.gsea-msigdb.org/gsea/msigdb) using the "c2.cp.kegg.v7.4.symbols.gmt" gene set, and to reflect the representativeness of the given gene set in the ranked gene list 18, 19.

### Immunoinfiltration and Immunotherapeutic Analysis

In this study, we employed single-sample gene set enrichment analysis (ssGSEA) to assess the abundance of various immune cell infiltrates and immune functional scores within each high and low expression group of HNSCC samples. The Immune Profile Score (IPS) showed a positive correlation with immune therapy response. IPS data for HNSCC patients were obtained from the Cancer Immunome Atlas (TCIA, https://tcia.at/home). Additionally, we downloaded Immunophenoscores (IPS) for HNSCC patients from the TCIA database (https://tcia.at/) 20, 21, which were used to predict the sensitivity to immune therapy.

### Drug Sensitivity Analysis

In this study, the "oncoPredict" package of R software was utilized to predict the IC50 values of various compounds for each high and low expression group of HNSCC samples. The screening threshold was set at p<0.001. The IC50 values were used as indicators of the potential of compounds to inhibit specific biological or biochemical functions.

### Prognostic Gene Selection and Risk Model Construction Methods

In this study, prognostic genes (p<0.05) were selected from DEGs based on univariate Cox regression analysis. Subsequently, the "glmnet" package in R software was utilized to perform Lasso-Cox regression for constructing the risk model. The "survivalROC" package in R software was employed to evaluate the predictive performance of high and low expression groups and to generate ROC curves and Kaplan-Meier (KM) survival curves.

### Column Line Chart Model Construction Method

We employed risk scores and clinical factors and constructed the column line chart model using the "rms" package in R software. The predictive capability of the column line chart model was assessed through the depiction of calibration curves and Decision Curve Analysis (DCA).

### Gene Interaction Analysis

We evaluated proteins that exhibit strong physical interactions with ECT2 based on the GeneMANIA database (http://www.genemania.org) 17. This database is used to generate hypotheses about gene functions, analyze gene lists, and prioritize genes for functional analysis.

### Collecting Patient Tissues and *In Vitro* Experiments in HNSCC Research

#### Patient and Sample Collection Methods

Patients diagnosed with HNSCC and undergoing surgical treatment at the Fourth Hospital of Hebei Medical University between September 2023 and March 2024 were selected for this study. Subject inclusion criteria: (1) Surgical specimens confirmed as HNSCC by histopathology; (2) Subjects had not received any tumor-related treatment (such as chemotherapy, radiotherapy or immunotherapy) prior to surgery; (3) Subjects had no serious heart, brain or lung disease and could tolerate surgery; (4) Subjects had no history of other malignancies. Subject exclusion criteria: (1) Subjects not fulfilling pathological diagnostic criteria; (2) Subjects who have received tumor-related treatment prior to surgery; (3) Subjects with serious heart, brain, lung or other diseases that cannot tolerate surgery; (4) Subjects with other malignancies, systemic infections, immune disorders, mental disorders, or serious metabolic diseases. Thirty patients' tissue samples were collected, including 30 samples of normal tissue and 30 samples of HNSCC tissue. Tissue specimens were collected during surgery, rapidly frozen in liquid nitrogen after excision, and then stored in a tumor specimen repository under frozen conditions until RNA was extracted for use. Paraffin-embedded tissues of HNSCC and normal control samples were retained from 15 patients in preparation for immunohistochemistry experiments. Each patient signed an informed consent form before participating in the study. The utilization of patient tissue samples received approval from the Ethics Committee of the Fourth Hospital of Hebei Medical University (Approval Number: 2020KY283).

#### Immunohistochemistry

The expression levels of ECT2 expression levels in HNSCC tissues were observed by immunohistochemistry. Tissue sections were placed in xylene for 10 min and immersed again for 10 min after changing xylene. After that, they successively went through absolute ethanol, 95% ethanol, and 75% ethanol for 5 min each. Then it was further incubated with 0.3% hydrogen peroxide for 15 min, microwave-repaired for 15 min, and cooled naturally. After blocking with 10% bovine serum albumin for 30 min, ECT2 antibody (1: 200, ab236502, Abcam, Cambridge, UK) was added. Overnight at 4°C and incubated with horseradish peroxidase-labeled secondary antibodies for 30 min. Finally, hematoxylin counterstaining and neutral gum mounting slides.

Two experienced pathologists independently observed all samples under a light microscope without knowledge of the patients. Immunoreactivity for ECT2 was evaluated using a combined scoring system based on intensity and extent. Staining intensity was categorized as follows: 0 for no staining, 1 for weak immunoreactivity, 2 for moderate immunoreactivity, and 3 for strong immunoreactivity. The percentage of cells with positive ECT2 staining was scored as follows: 0 for ≤5% positive cells, 1 for 6-25% positive cells, 2 for 26-50% positive cells, 3 for >50% positive cells. The final scores were calculated by combining the staining intensity score and the positive percentage score. Scores of ≤3 was considered as low expression, while scores of >3 was regarded as high expression.

#### Cell Cultivation and Transfection

The human HNSCC cell line HSC-3 was obtained from the biological specimen bank of the Fourth Hospital of Hebei Medical University. The HNSCC cell lines were cultured in DMEM medium (Procell, Wuhan, Hubei, China), supplemented with 10% Fetal Bovine Serum (FBS), 100 U/mL penicillin, and 100 μg/mL streptomycin. Incubation was maintained at 37 °C, with 5% CO_2_ concentration and optimal humidity. si-ECT2 [*#1*:5'-CCUUGUAGUUGAAGAGAAUTT-3'; antisense: 5'-AUUCUCUUCAACUACAAGGTT-3'. *#2*: 5'-GGCAACAAUUAUUCAGUUATT-3'; antisense: 5'-UAACUGAAUAAUUGUUGCCTT-3'] and its corresponding negative control (si-NC: 5'-UUCUCCGAACGUGUCACGUTT-3') were procured from Sangon Biotech Co., Ltd (Shanghai, China). Transfection was carried out when the cell density reached approximately 70%~80%. Lipofectamine 2000 reagent (Invitrogen, Carlsbad, CA, USA) was employed following the protocol. Transfection efficiency was estimated through quantitative real-time polymerase chain reaction (qRT-PCR) after 24 hours of incubation.

#### qRT-PCR

Trlquick Resgent (Solarbio, Beijing, China) reagent was employed for RNA extraction from tissues and cells. Subsequently, the reverse transcription and cDNA synthesis were carried out using HiScript III RT SuperMix for qPCR (+gDNA wiper) (Vazyme, Nanjing, China). Following this, Hieff® qPCR SYBR Green Master Mix (No Rox) (Yeasen, Shanghai, China) was utilized for qRT-PCR to assess mRNA expression levels. Primers used were provided by Sangon Biotech (Shanghai, China). GAPDH served as an internal reference, and the relative expression levels of mRNA were calculated using the 2^-ΔΔCt^ method. Primers for qRT-PCR (5′→3′): ECT2: Forward, AAGAGTGGTTCTGGGGAAGC; Reverse, TGCGATTGCTGTTAGGGGTA. GAPDH: Forward, GAAGGTGAAGGTCGGAGTC; Reverse, GAAGATGGTGATGGGATTTC.

#### Cell Counting Kit-8 (CCK-8) Assay

The cells that underwent transfection were introduced into 96-well plates at a density of 1.5×10^3^ cells per well. These cells were then further cultivated for intervals of 0 hour, 24 hours, 48 hours, and 72 hours. Once each respective time point was reached, we introduced 10 μl of CCK-8 reagent (MedChemExpress, Monmouth Junction, NJ, USA) to each well, followed by a 2 hours incubation period. Subsequently, we employed a microplate reader (BioTek, Winooski, VT, USA) to measure the optical density (OD) at 450 nm for each well.

#### Cell Migration and Invasion Assays (Transwell)

After 24 hours of transfection, the cells were resuspended in serum-free medium at a concentration of 3×10^5^ cells/ml. Then, 200 μl of this cell solution was withdrawn and introduced into the upper compartment of a Matrigel matrix gel-coated transwell (Corning, Corning, NY, USA). The lower chamber was supplied with culture medium with 10% FBS. The cells were then cultured for 24 hours in the incubator. Cotton swabs were used to remove the cells in the upper chamber, followed by fixation with 4% paraformaldehyde and staining with 0.5% crystal violet. Photomicrographs were captured using a microscope (Mshot, Guangzhou, Guangdong, China). In the migration assay, no matrix glue was added.

#### Cell Apoptosis Assay

When transfection was done, cells were located into 6-well plates (5×10^5^ cells/well) and cultivated for 48 hours. Following two washes with PBS, the cells were got by centrifugation. As per the instructions outlined in the Annexin V-FITC/PI apoptosis detection kit (Keygen, Nanjing, Jiangsu, China), the cells were resuspended in 500 μl binding buffer. Then, 5 μl of Annexin V-FITC was introduced and thoroughly mixed, afterward adding of 5 μl of PI and another round of mixing. The cells were incubated at room temperature, shielded from light, for a duration of 5~15 minutes. Flow cytometry (Beckman, Indianapolis, IN, USA) was employed to assess apoptosis.

#### Cell Cycle Assay

Following the transfection, cells were planted into 6-well plates at a concentration of 5×10^5^ cells per well and cultured for 48 hours. They underwent two rinses with PBS, were collected, and resuspended. The cells were then fixed in 70% chilled ethanol at 4°C for more than 4 hours. Centrifugation was used to gather the cells. After a chilled PBS wash, 200 μl of PBS was employed for resuspension. To this, 10 μl of RNase (1mg/ml; Beyotime, Shanghai, China) was used, and the mixture was incubated at 37°C for 30 minutes. Subsequently, 10 μl of PI (400 μg/ml; Beyotime, Shanghai, China) was introduced, and the cells were stained in ow light conditions at 4°C for 30 minutes. Cell cycle distribution was evaluated using flow cytometry (Beckman, Indianapolis, IN, USA).

#### Statistical Analysis

Data analysis was executed with GraphPad Prism (v10.0; GraphPad Software, La Jolla, CA, USA). The results were expressed as the mean ± standard deviation (Mean ± SD). Wilcoxon rank sum test was used for expression differences in tissue samples. One-way ANOVA + multiple hypothesis test (Tukey HSD post hoc test) was used for transfection efficiency, apoptosis, cycle, migration, and invasion assay. TWO-way ANOVA was used for CCK-8 assay. The significance threshold was established at p<0.05. It's important to note that three times of experiments were replicated for robustness and reliability.

### Analysis Methods of scRNA-seq Data

The workflow for the analysis of scRNA-seq data is as follows. The original data files are loaded using the Read10X function in the "Seurat" package 22 of the R software. Each sample is processed separately, and the CreateSeuratObject function is used to create a Seurat object for each sample. Only cells with at least 3 detected genes are retained for further analysis. Cells are filtered using the subset function in the Seurat package based on the number of detected features and the percentage of mitochondrial genes. The dataset is normalized using the LogNormalize method, and then variable features are identified using the FindVariableFeatures function. Principal component analysis (PCA) is performed using the RunPCA function. Subsequently, the "DoubletFinder" package 23 in R software is used to detect cell doublets after parameter optimization using sweep statistics from scRNA-seq data, with the doubletFinder function. The "SingleR" package 24 in R software annotates single-cell transcriptomes based on a reference database of human cell types. The SingleR function assigns cell types to individual cells based on their transcriptomic features. Dimensionality reduction technique based on t-distributed stochastic neighbor embedding (t-SNE) is performed sequentially using the RunTSNE function and DimPlot function to visualize all cells in two dimensions. The "AUCell" package 25 in R software is used for gene set enrichment analysis to assess the activity of predefined gene sets among different cell types. Differential gene expression analysis between different cell types is performed using the FindMarkers function in the Seurat package. Finally, the "CellChat" package 26 in R language evaluates significant pathways of interaction between different types of cells.

### Mendelian Randomization Analysis Method

In this investigation, we utilized the R package TwoSampleMR (v0.5.7) to evaluate the causal association between genes and Head and neck cancer. To ensure the independence of single nucleotide polymorphisms (SNPs), we conducted clustering and excluded SNPs with a linkage disequilibrium (LD) r² < 0.001. The genome-wide significance threshold for traits was set at (p < 5 × 10⁻⁸). We evaluated the instrumental strength of each SNP using the F-statistic, and reported the average F-statistic for SNPs acting as instruments, with F-statistic > 10 indicating robust instruments 24. For a single instrument, the Wald ratio test was employed by calculating the Wald estimate, obtained by dividing the SNP outcome by SNP exposure. In the case of multiple instruments, the Inverse Variance Weighted (IVW) method was applied, incorporating information from all instruments. Causal relationships were considered statistically significant if corrected p-values were less than 0.05. Heterogeneity was assessed in IVW estimates, where high heterogeneity suggests substantial variance among different instruments, indicating ineffective instruments. To visually represent Mendelian Randomization (MR) results, scatter plots depicting the influence of SNPs on exposure versus outcomes were generated. Forest plots were utilized to present estimates from multiple instruments. Funnel plots were created for an intuitive assessment of heterogeneity, and MR estimates were visualized when omitting one instrument.

### Statistical Analysis

Data analysis and statistical analysis in this study were conducted using R software (version 4.2.1). The Wilcoxon signed-rank test was employed to examine the relationship between clinical parameters and ECT2, the differential expression of ECT2 between the diseased group and the control group, as well as differences in immune cells, immune therapy, and drug sensitivity between high and low expression groups of ECT2. Spearman's correlation analysis was used to explore the correlation between prognostic genes and immune cells, as well as the correlation between interacting genes and immune cells. Kaplan-Meier (KM) log-rank test was utilized for survival analysis. A P-value < 0.05 was considered statistically significant.

## Results

### Reduced ECT2 Expression Altered the Phenotype of HSC-3 cells

**Figure 1:**
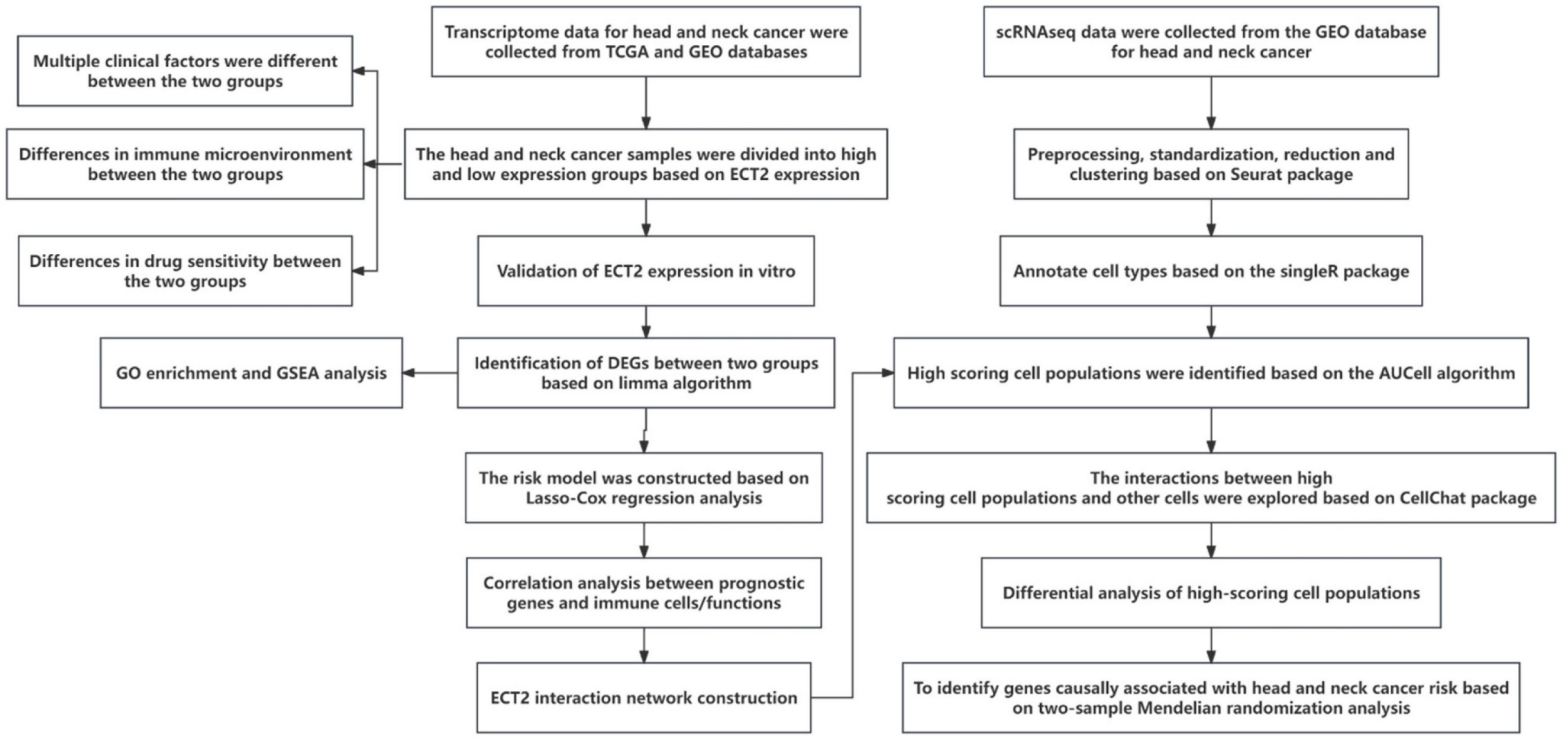
The technical roadmap.

**Figure 2:**
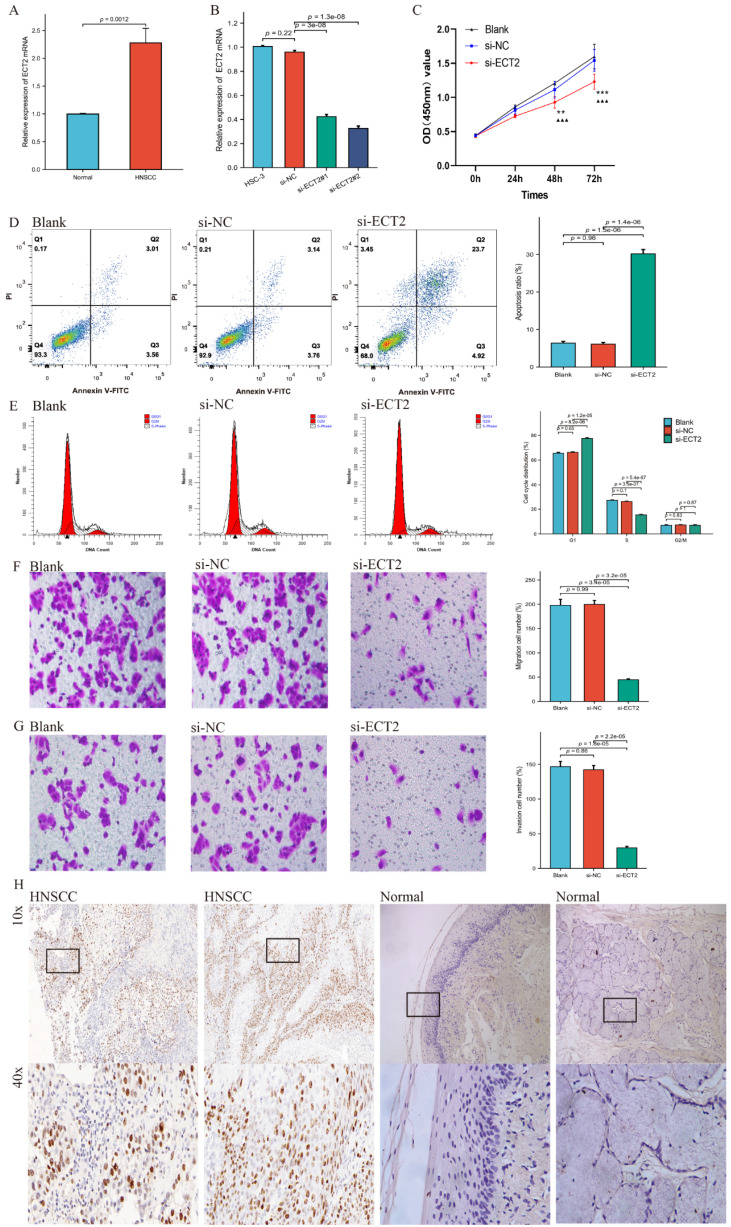
Reduced ECT2 expression altered the phenotype of HSC-3 cells. (A) The expression level of ECT2 in HNSCC samples and normal samples (30 patients) was analysed using qRT-PCR, with GAPDH as an internal reference. (B) Si-ECT2#1, si-ECT2#2, and si-NC were transfected into HSC-3 cells. (C) Cell viability was detected using a CCK-8 assay (Blank vs si-ECT2, ▲▲▲ P<0.001; si-NC vs si-ECT2, ** P<0.01, *** P<0.001). Apoptosis assay (D) and cell cycle assay (E) were performed 48 h after transfection. Transwell assay for cell migration (F) and invasion (G), x200. (H) Immunohistochemical analysis of ECT2 expression in HNSCC and normal samples.

Figure 1 presents the overall flow chart of this paper. The qRT-PCR assay showed that the expression of ECT2 in HNSCC samples was significantly higher than in normal samples (Figure 2A). Immunohistochemical results analysed by Fisher test showed that the expression of ECT2 was significantly higher in the HNSCC group (13/15) than in the control group (6/15; p = 0.021) (Figure 2H). Furthermore, based on the TCGA-HNSC cohort, we analyzed the differential expression of ECT 2 between normal and HNSCC. The results showed that in the TCGA-HNSC cohort, the expression of ECT2 was significantly higher in the HNSCC group than in the normal group, further confirming the above results (Figure S1). To investigate the biological function of ECT2 in HNSCC, we transfected si-ECT2#1, si-ECT2#2, and si-NC into HSC-3 cells. The most significant reduction of ECT2 expression was observed in the si-ECT2#2 group (Figure 2B). Therefore, we selected si-ECT2#2 for subsequent experiments. Compared to the si-NC group, HSC-3 cells transfected with si-ECT2#2 exhibited reduced viability (Figure 2C), increased apoptosis (Figure 2D), and an increased proportion of cells in G1 phase, with a decreased proportion in S phase (Figure 2E). The results indicate that the reduction of ECT2 expression inhibited the proliferation of HNSCC cells, induced apoptosis, and impeded the progression from G1 to S phase *in vitro*. The effects of ECT2 on HNSCC cell migration and invasion were subsequently assessed. The migration (Figure 2F) and invasion (Figure 2G) abilities of HSC-3 cells were inhibited after transfection with si-ECT2#2 compared to the si-NC group, as expected. These results suggest that changes in ECT2 expression regulate the phenotype of HNSCC cells. Finally, we present the boxplots of ECT2 expression in the transcriptome data of the control and HNSCC groups. The expression of this gene was significantly different between the two groups (Figure S1).

### Basic Analysis Results of ECT2-Related Genes

**Figure 3:**
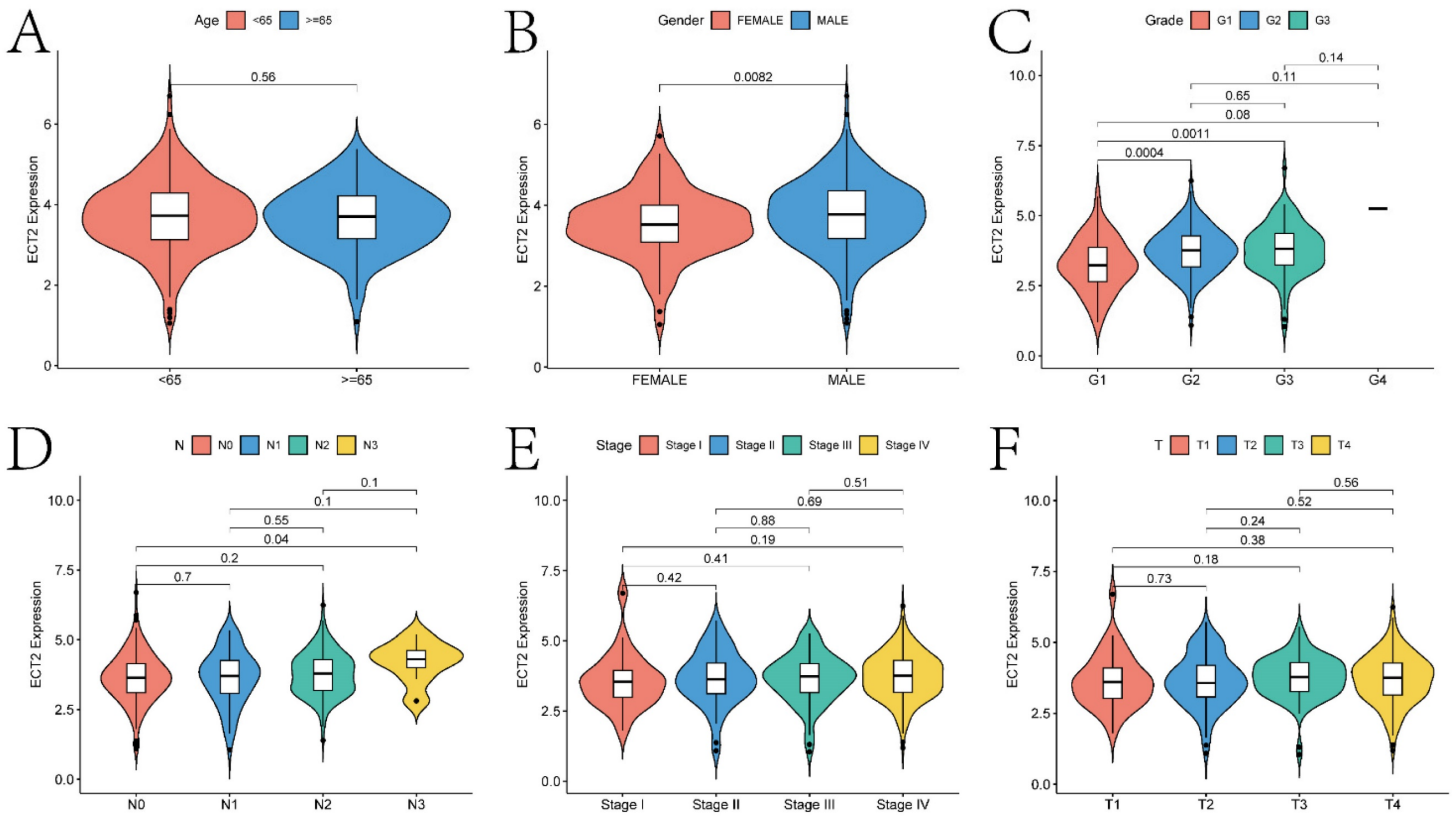
The correlation analysis between ECT2 expression and clinical prognostic indicators. Violin plots A-F show the correlation between ECT2 expression and age, gender, Grade, N staging, Stage staging, and T staging, respectively.

The basic analysis results of ECT2-related genes indicate significant differences in the expression of ECT2 concerning various clinical indicators (Figure 3; gender, age, grade classification, T stage, N stage, and stage classification). Specifically, significant differences (p < 0.05) in ECT2 expression were observed in the gender, G1 and G2, G1 and G3, as well as N0 and N3 groups. The ECT2 mRNA expression levels and clinicopathological features of TCGA-HNSC cohort (511 patients) and self-selected cohort (30 patients) in the study were further correlated.

The median value of ECT2 mRNA expression level was used to classify into two groups of high and low expression. The results similarly showed that the dysregulation of ECT2 expression was correlated with the Gender or Grade classification of HNSCC patients (p < 0.05) (Table 1-2). The above results show the potential value of ECT2 in the clinical setting.

### Analysis of ECT2 High and Low Expression Groups

**Figure 4:**
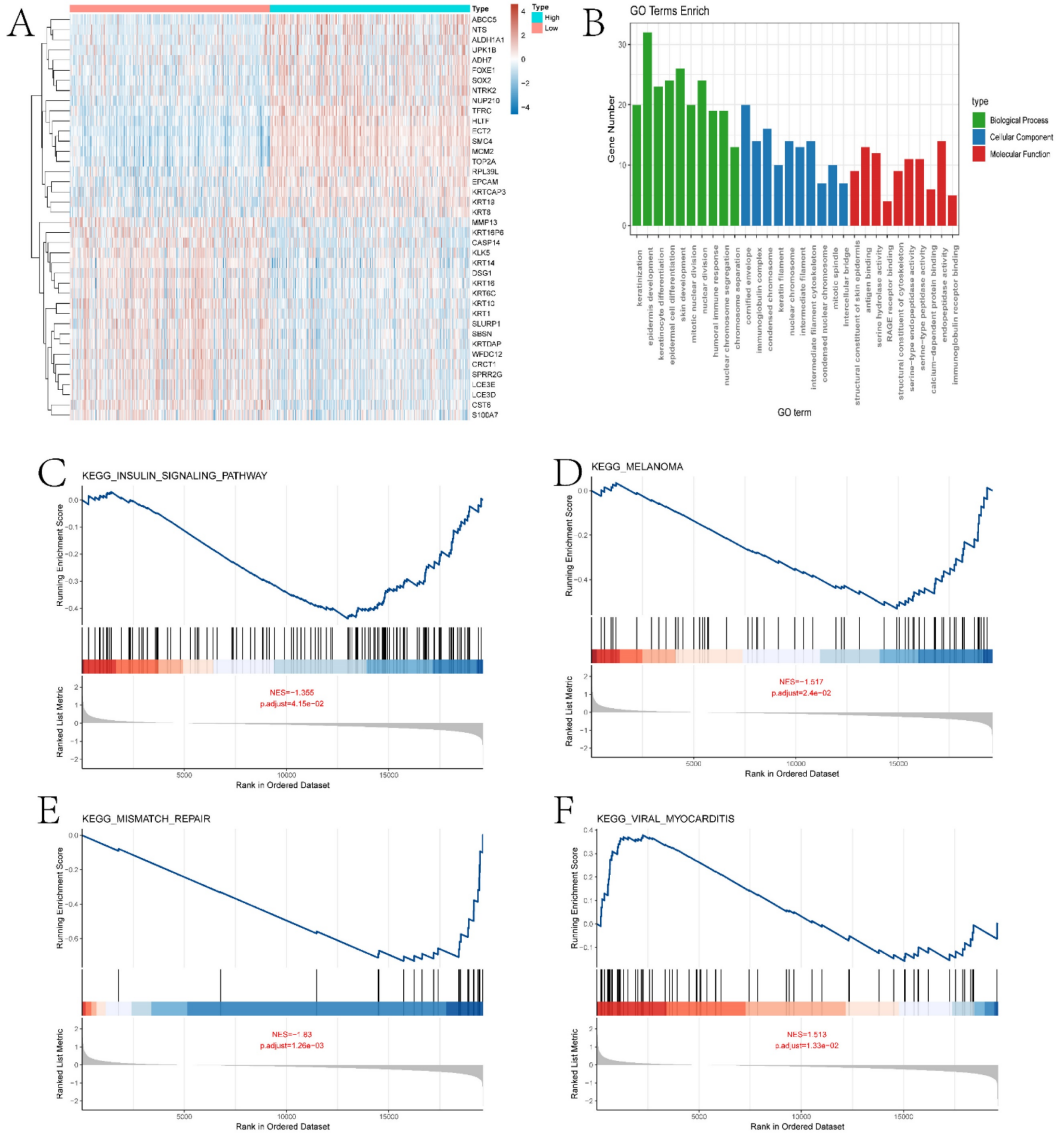
Enrichment analysis results of ECT2-related genes with high and low expression levels. A shows the expression heatmap of DEGs (Differentially Expressed Genes) between high and low expression groups of ECT2. B presents the GO enrichment analysis results of DEGs. C-F display the GSEA (Gene Set Enrichment Analysis) results of high and low expression groups of ECT2.

**Figure 5:**
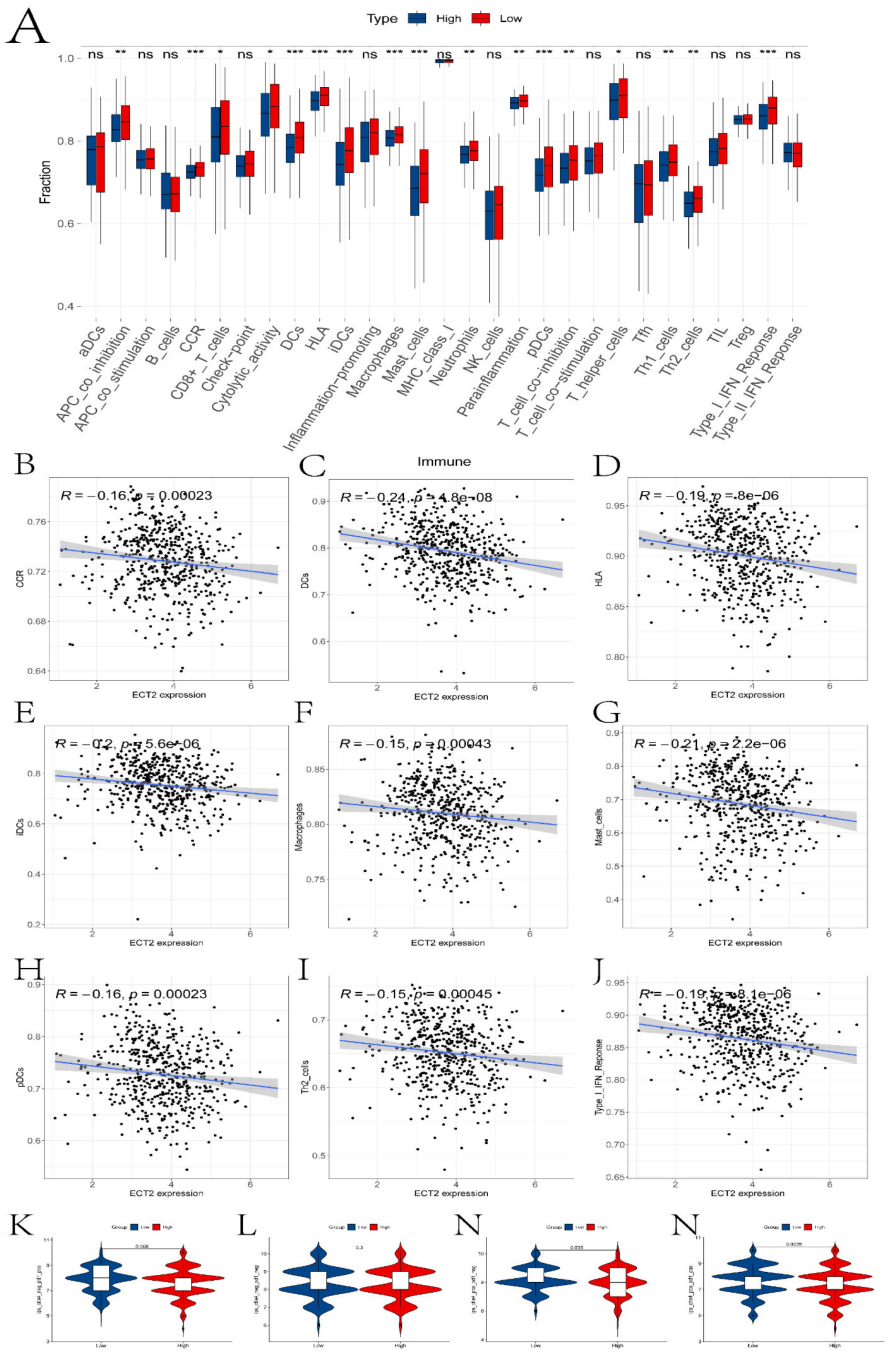
Analysis of the immune microenvironment between high and low expression groups. A presents boxplots illustrating differences in various immune functional scores and immune cell infiltration abundance between high and low expression groups. B-J depict correlation analyses between ECT2 and immune cells. K-N show the results of immune therapy analysis between high and low expression groups.

In this section, we performed a detailed analysis of the DEGs between the high and low expression groups, as well as the differences between the two groups in terms of immune microenvironment and drug sensitivity. Specifically, to explore the DEGs between the high and low expression groups defined by ECT2, we identified 232 DEGs based on the limma algorithm. Figure 4A presents the differential expression heatmap of the top 40 DEGs in both groups. GO enrichment analysis of DEGs (Figure 4B) and GSEA analysis between the two groups identified multiple pathways associated with the occurrence and development of HNSCC. Figures 4C-F display some pathways, and the remaining pathways are provided in the "GSEA folder" in the supplementary materials. We will discuss in detail the close relationship between these pathways and HNSCC in the Discussion section. Subsequently, we evaluated the immune microenvironment of HNSCC samples using the ssGSEA algorithm (Figure 5A). We will analyze in detail the significantly different immune cell infiltration abundance and immune function scores between the two groups in the Discussion section. ECT2 shows significant correlations with various immune cells/functions (Figure 5B-J; CCR, DCs, HLA, iDCs, Macrophages, Mast cells, pDCs, TH2 cells, and TypeⅠIFN response).

**Figure 6:**
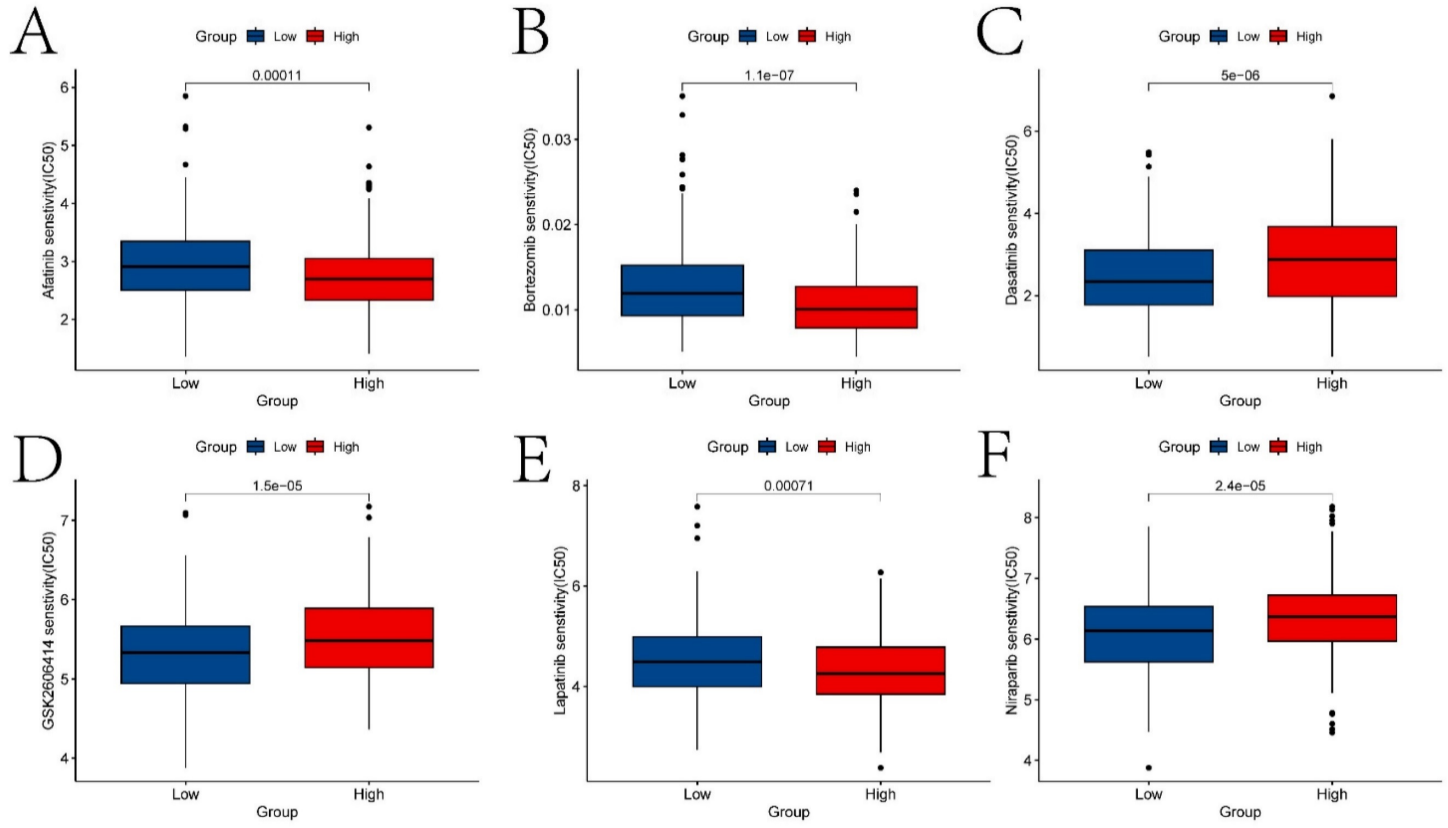
The analysis results of drug sensitivity in ECT2 high and low expression groups. Violin plots (A-F) depict drugs with significant differences in IC50 values between high and low expression groups.

Previous studies have reported the role of Immune Prognostic Score (IPS) based on immunogenicity in predicting the immune therapeutic response in cancer patients. We analyzed the relationship between high and low expression group samples and IPS. We used IPS to evaluate the potential of the two groups to respond to immune therapy. ips_ctla4_neg_pd1_neg indicates no response to CTLA-4 and PD-1 antibodies; ips_ctla4_neg_pd1_pos indicates no response to CTLA-4 and response to PD-1; ips_ctla4_pos_pd1_neg indicates response to CTLA-4 and no response to PD-1; ips_ctla4_pos_pd1_pos indicates response to both CTLA-4 and PD-1 antibodies. From Figure 5K-N, it can be observed that except for ips_ctla4_neg_pd1_neg, the other conditions show significant differences between the two groups. This suggests that the high and low expression groups defined by ECT2 have great potential in predicting prognosis and immune therapeutic benefits. Finally, we also evaluated the differences in IC50 values of various compounds between the two groups (Figures 6A-F and the “drug folder” in the supplementary materials).

### Selection of Prognostic Genes and Construction of Risk Models

**Figure 7:**
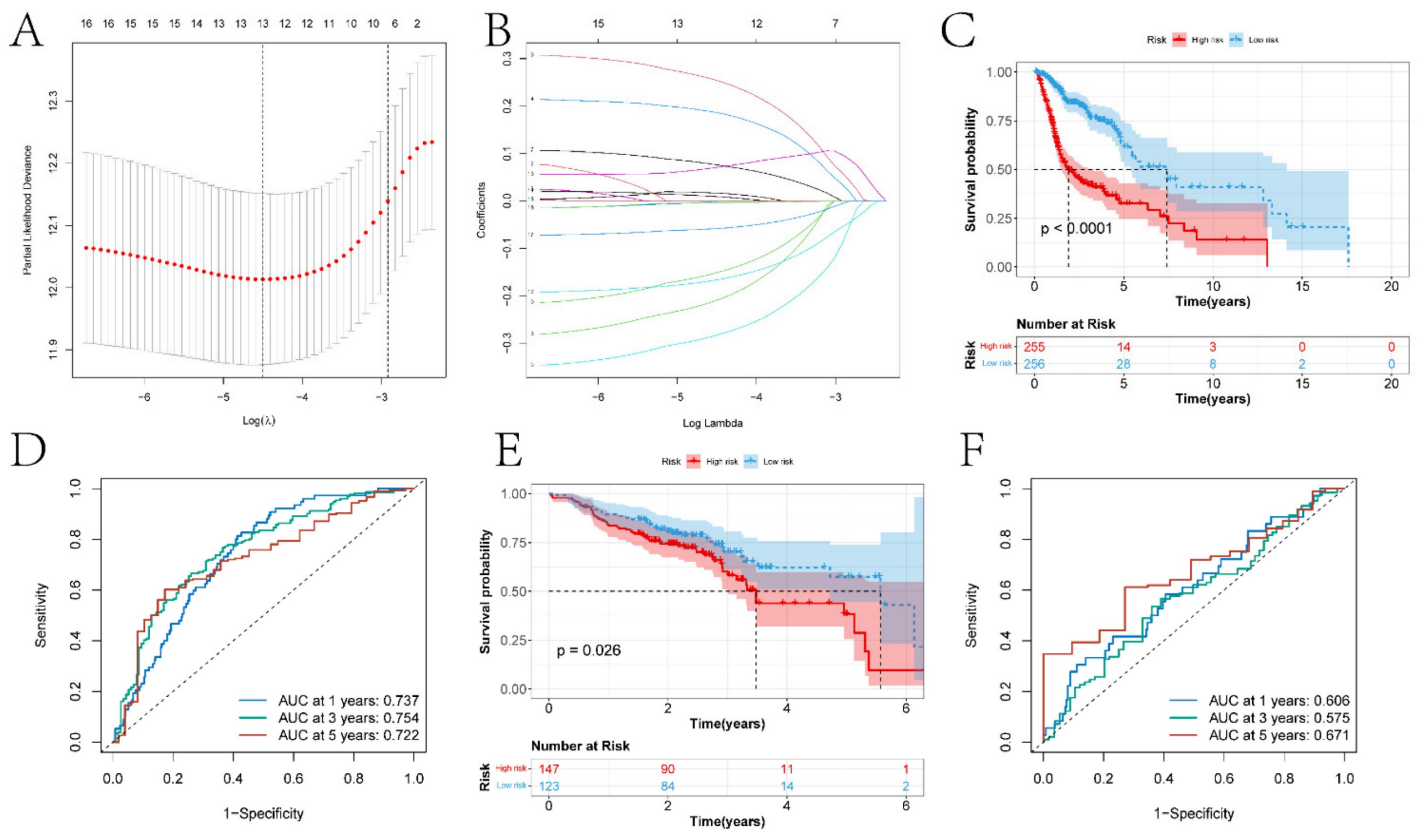
The construction of risk models and the differences in prognosis among different expression groups. A-B respectively represent the variation characteristics of the Lasso variable coefficients and the optimal values of parameter λ selected through cross-validation methods in the Lasso regression model. C and E respectively represent the KM survival curves of the training and testing sets. D and F respectively represent the ROC curves predicting the 1-year, 3-year, and 5-year survival of high and low-risk group patients in the training and testing sets.

In this study, previously identified DEGs were subjected to univariate Cox regression analysis to screen prognostic genes (Figure S2), resulting in a total of 17 genes being identified (ANLN, FOXRED2, ADD3, DSG2, PTPRS, FOXE1, CDA, TXNRD1, FADS2, FSTL3, CNFN, SPINK6, MT2A, MT1E, SPINK7, PTN, and DEFB1). Furthermore, a risk model was constructed through Lasso-Cox regression analysis (Figure 7A-B). The differences in prognosis between the high and low-risk groups of samples divided by the risk model in the training and testing sets were investigated. KM survival analysis on both datasets indicated significant differences in survival between the high and low-risk groups (p < 0.05) (Figures 7C and 7E). In the training set, the model predicted AUCs for patient survival at 1 year, 3 years, and 5 years were 0.737, 0.754, and 0.722, respectively (Figure 7D). In the testing set, the model predicted AUCs for patient survival at 1 year, 3 years, and 5 years were 0.606, 0.575, and 0.671, respectively (Figure 7F).

### Construction of the Line Graph Model and Results of Gene Interaction Analysis

**Figure 8:**
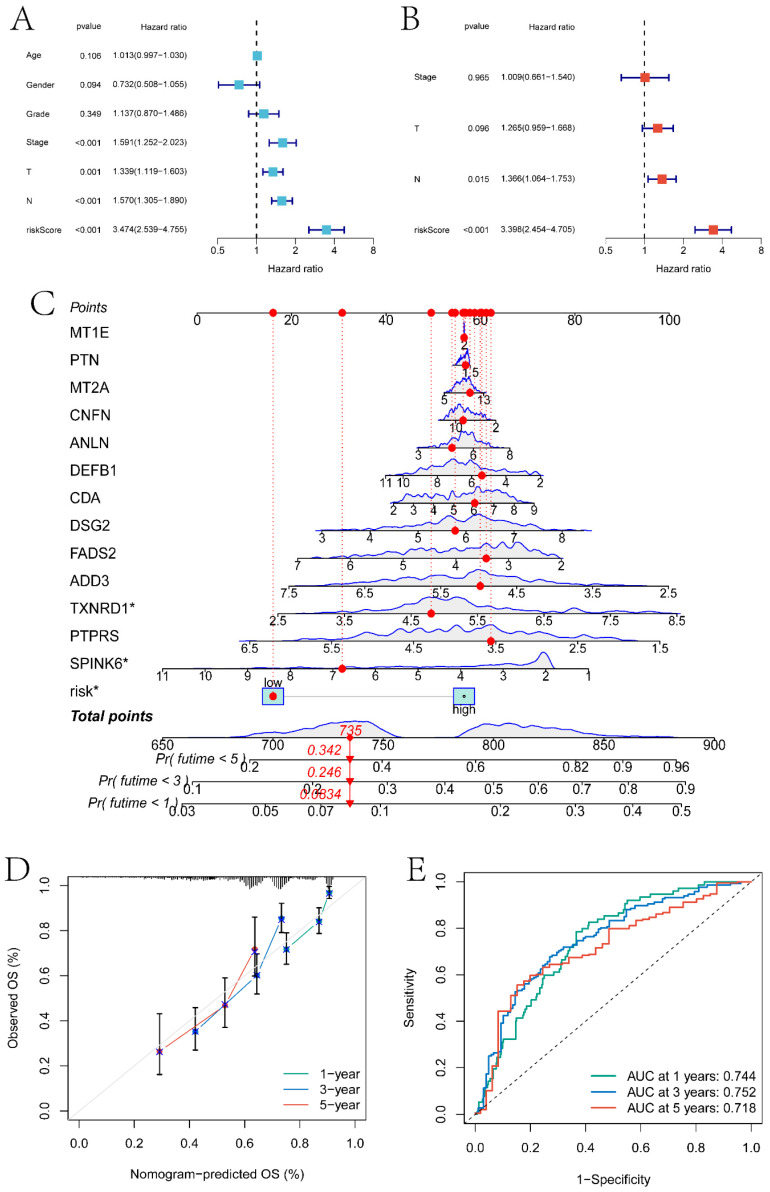
Independent prognosis analysis and nomogram construction. A and B respectively are single-factor Cox regression analysis and multi-factor Cox regression analysis based on clinical factors and risk score to screen independent prognostic factors. C is the nomogram model constructed based on independent prognostic factors. D is the calibration curve of the nomogram model. E is the ROC curve of the nomogram model predicting the survival status of patients at 1 year, 3 years, and 5 years.

In this section, we combined clinical factors and risk scores to screen for independent prognostic factors in patients. Firstly, through univariate Cox regression analysis, risk scores, Stage, T stage, and N stage were found to be significantly correlated with patient prognosis (p<0.05) (Figure 8A). Subsequently, through multivariate Cox regression analysis, N stage and risk scores were identified as independent prognostic factors (p<0.05) (Figure 8B). Using these features, a line graph model was constructed (Figure 8C), and the calibration curve indicated that the model had a high predictive ability (Figure 8D). The model predicted the AUC of patient survival at 1 year, 3 years, and 5 years to be 0.744, 0.752, and 0.718 respectively (Figure 8E).

### Analysis Results of scRNA-seq and Mendelian Randomization

**Figure 9:**
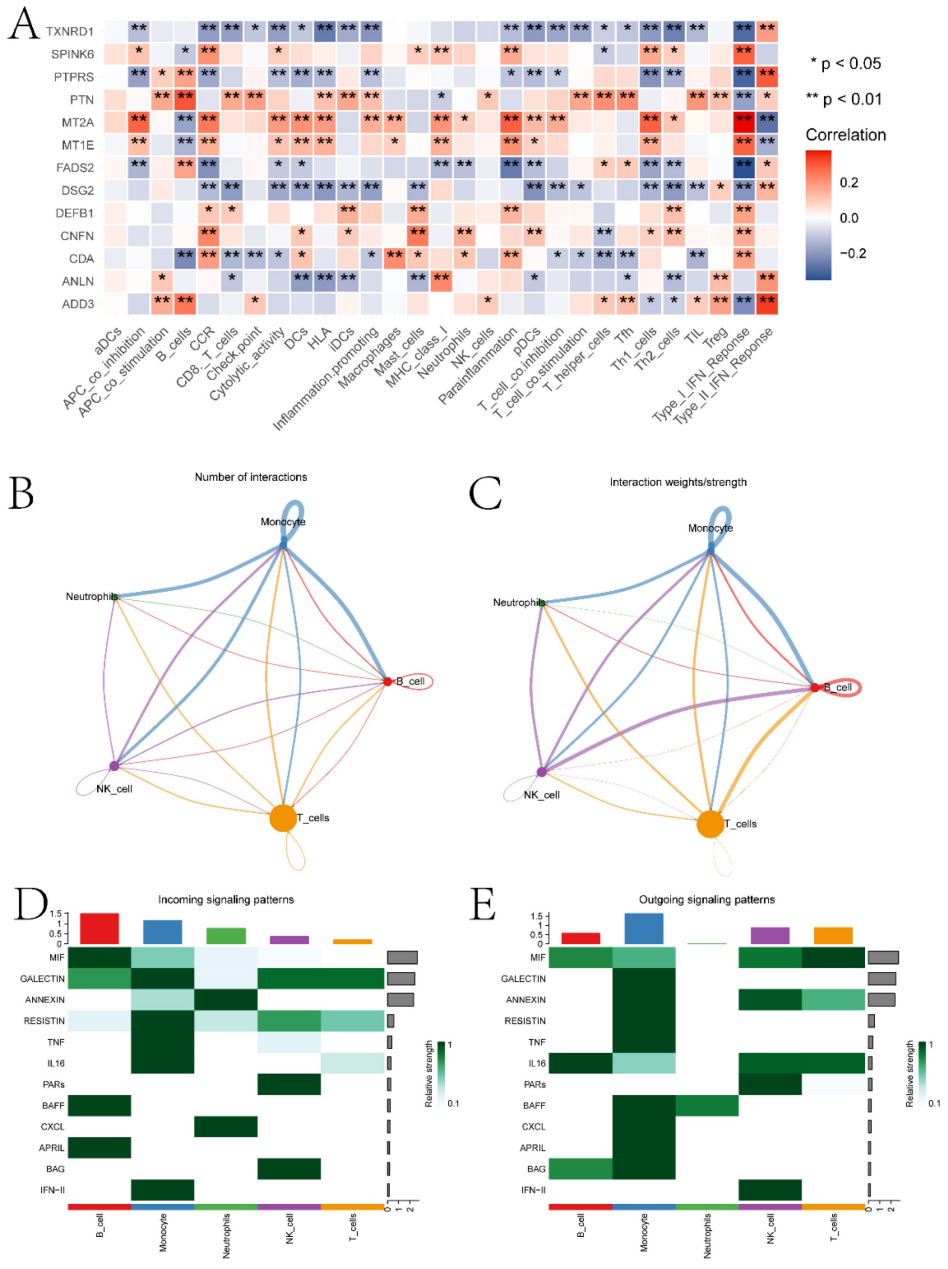
Immunoreactivity analysis and Cell communication analysis results. A is the correlation heatmap between prognostic genes and immune cells. B and C are network diagrams showing the quantity and weight of interactions between different types of cells, respectively. D and E are pathway heatmaps illustrating the interactions between different types of cells as receptors and ligands, respectively. (* P<0.05, ** P<0.01, *** P<0.001).

Using the singleR package, five cell types including Monocytes, T cells, NK cells, B cells, and dendritic cells were annotated (Figure S3A-B). Further exploration was conducted on the expression of ECT2 and genes interacting with it in each type of cell population (Figure S3C). RHOA showed high expression in the Monocyte population. MRPL27 and UBE3A were highly expressed in the dendritic cell population. The scoring of different cell types was evaluated based on the AUCell algorithm, with the Monocyte cell population receiving the highest score (Figure S3D). We evaluated the correlation between prognostic genes and the abundance/immune function score of immune cell infiltration and the correlation between prognostic genes (Figure 9A). Most prognostic genes showed significant correlations with the abundance/immune function score of immune cell infiltration. To explore the expression landscape of ECT2 and its interacting genes in scRNA-seq data, scRNA-seq data of HNSCC samples were downloaded from the GEO database. We further explored the top 10 pathways based on the AUCell algorithm scores (Figure S4). Finally, through cell communication analysis, strong communication relationships between the Monocyte population and other cell populations were discovered (Figure 9B-C). Pathways of interaction between different cell types acting as receptors and ligands were also investigated (Figure 9D-E). In the discussion section, we will provide a detailed analysis of the top 10 pathways and the relationship between the Monocyte cell population and other cell signaling pathways in HNSCC.

**Figure 10:**
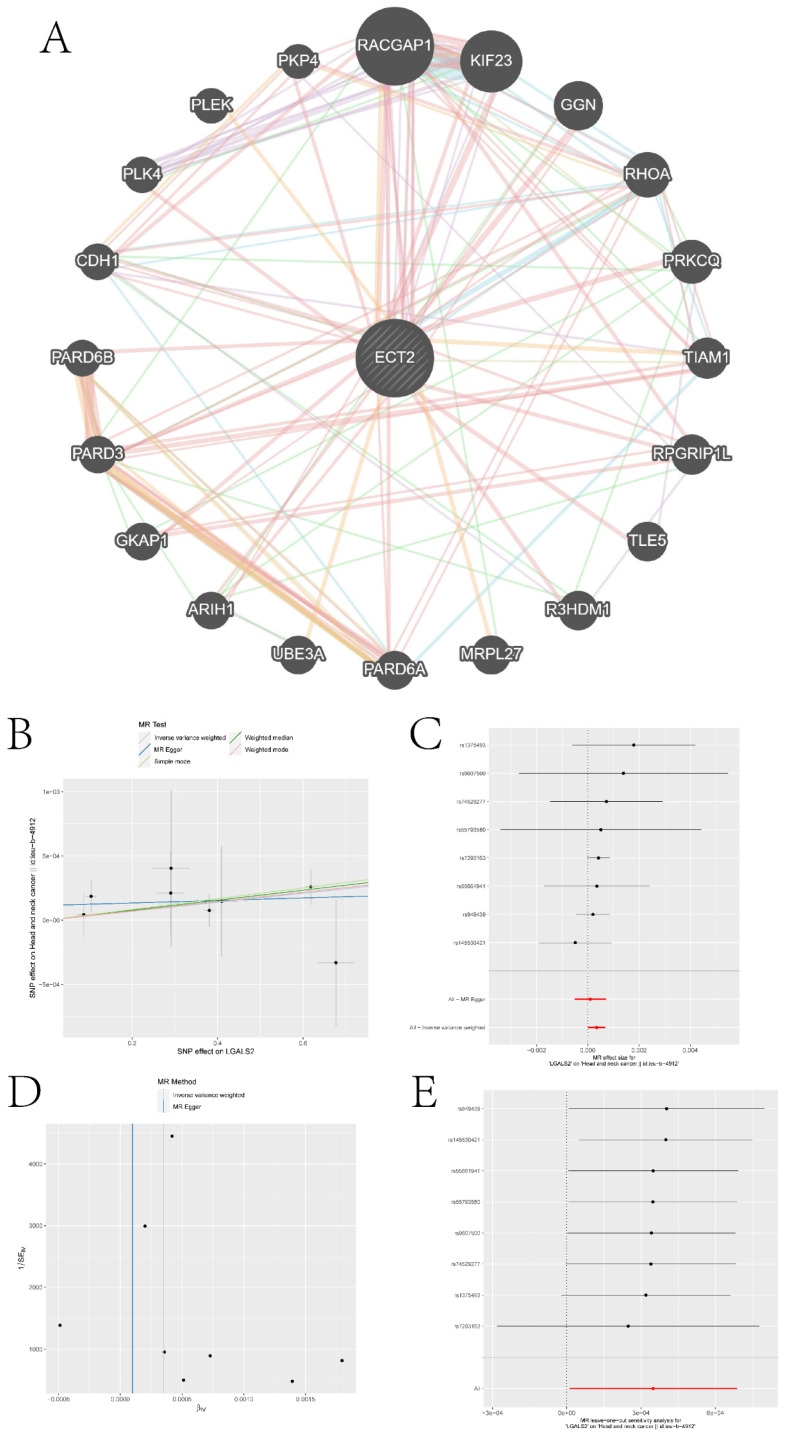
Construction of gene interaction networks and the MR analysis results of the causal relationship between LGALS2 and HNSCC. A is the gene-gene interaction network constructed based on Genemania data. B represents the influence of SNPs on HNSCC among five models. C denotes the causal relationship of LGALS2 on AD represented by the Wald ratio. D and E present the results of sensitivity analysis.

We queried the Genemania database for genes that interact with ECT2 and constructed an interaction network between ECT2 and other genes (Figure 10A). Mendelian randomization (MR) analysis revealed that the expression of three genes, LGALS2, SLC11A1, and TKT, among the DEGs between the Monocyte group and other cell groups, is causally associated with the risk of HNSCC. Both the weighted median model and IVW model supported the causal relationship between LGALS2 and HNSCC. Detailed results of the causal relationship analysis between LGALS2 and HNSCC risk are shown in Figure 10B-E. As depicted in Figure 10B, the linear slopes of all five models are greater than 0, indicating a positive correlation between exposure and outcome. The forest plot in Figure 10C suggests that both MR Egger and IVW methods support that this gene is a risk factor for HNSCC. Funnel plot results indicate no directional horizontal pleiotropy affecting IVW and weighted median estimates (Figure 10D). The orest plot derived from leave-one-out sensitivity analysis in Figure 10E demonstrates that the causal relationship is not driven by any single SNP with strong influence. Detailed information on the other two genes is provided in the supplementary materials (Figure S5-S6). SLC11A1 is a risk factor for HNSCC, while TKT serves as a protective factor against HNSCC.

## Discussion

This study delves into the potential role of ECT2 in head and neck squamous cell carcinoma (HNSCC) by integrating transcriptomic and single-cell RNA sequencing (scRNA-seq) data. Additionally, Mendelian randomization analysis is employed to explore genes causally associated with HNSCC. Firstly, based on the expression of ECT2, all HNSCC samples were divided into high and low expression groups, and the DEGs between the expression groups and the biological pathways they participate in were explored. Such as serine-type endopeptidase activity and epidermal cell differentiation. In some studies identifying molecular events in the progression of head and neck squamous cell carcinoma, a novel serine protease DESC1 was isolated, and calcium-induced normal keratinocyte differentiation was accompanied by increased DESC1 expression 27. Additionally, the lineage-dependent oncogene TP63 in squamous cell carcinoma plays a significant role in the process of epidermal differentiation 28. Some top pathways involving high and low expression groups have also been confirmed to be closely related to HNSCC. For example, it has been shown in literature that WEE1 inhibitors are being studied in preclinical trials to enhance mitotic catastrophe and cell death for the treatment of head and neck squamous cell carcinoma 29.

Second, this study constructed a risk model for HNSCC based on DEGs. The risk model built through Lasso-Cox regression analysis consists of 17 prognostic genes: ANLN, FOXRED2, ADD3, DSG2, PTPRS, FOXE1, CDA, TXNRD1, FADS2, FSTL3, CNFN, SPINK6, MT2A, MT1E, SPINK7, PTN, and DEFB1. Among them, ANLN, DSG2, TXNRD1 and CNFN have been confirmed to be closely related to HNSCC. The abnormal expression and functional abnormalities of ANLN may be associated with the occurrence and development of HNSCC 30. Research has shown that the subtype of ANLN selective splicing exhibits synergistic effects *in vitro* and *in vivo*, promoting tumorigenesis of HNSCC 31. DSG2 is a cell-cell adhesion protein involved in intercellular adhesion. Literature has identified the core protein subtype switch in squamous cell carcinoma of the head and neck, with DSG2 being an important metric.

Morris *et al.* found that gene deletions of the EGFR phosphatase PTPRS frequently occur in HNSCC and can serve as important targets for HNSCC therapy 32. *In vitro* experiments indicate that knocking out TXNRD1 can inhibit the malignant activity of HNSCC cells 33. CNFN may play a role in the progression and metastasis of HNSCC through the EMT pathway 34.

Third, differences in the immune microenvironment between high and low expression group samples were explored using the ssGSEA algorithm. In the boxplot depicted in Figure 5A, we investigated the relationship between several immunocytes/functions significantly correlated with HNSCC (including CCR and its related chemokines, HLA and macrophages). In HNSCC, CCR and its associated chemokines may be involved in regulating the tumor immune environment, inflammatory responses, and tumor cell migration. For instance, the overexpression of has-miR-125a-5p enhances the proliferation, migration, and invasion of HNSCC cell lines by upregulating CCR7 35. HLA plays a crucial role in antigen presentation and immune responses and can serve as a prognostic marker for HNSCC 36. The activities of macrophages in the tumor microenvironment can significantly impact tumor development, immune evasion, and treatment response. Studies have found that tumor-associated macrophages play an important prognostic role in HNSCC 37. Additionally, differences in the IC50 values of various compounds were explored in two sample groups (Figure 6). Ferrarotto *et al.* evaluated the efficacy and tolerability of Afatinib in patients with HNSCC 38. Bortezomib inhibits protein degradation in the proteasome, blocking some key cellular signaling pathways. These pathways play crucial roles in the survival, proliferation, and drug resistance of cancer cells. Boribong *et al.* 39 described how bortezomib induces apoptosis in HNSCC cells by inhibiting Akt activation mediated by protein phosphatase 2A (CIP2A), making it an effective drug choice for treating HNSCC. Data suggest that Dasatinib treatment holds promise as a therapeutic option for DDR2 overexpressing HNSCC patients 40.

Fourth, this study investigates the expression landscape of ECT2 and its related genes in different types of cells based on scRNA-seq data. The Monocyte cell population was identified as a high-scoring cell group using the AUCell algorithm. The pathways of interaction between this cell group and other cell groups were explored. Monocytes are a type of white blood cell that play crucial roles in immune responses, particularly in inflammation and immune regulation. Chronic inflammation is associated with the development and progression of HNSCC. Inflammation may promote tumor formation and invasion through various pathways. The activity of mononuclear cells may play a significant role in this process 41. Studies indicate that B cells may participate in immune surveillance by producing antibodies, and B cells could represent a promising new direction for HNSCC therapy, allowing HNSCC patients to benefit from an immunotherapeutic perspective 42. Research on Monalizumab primarily focuses on the treatment of solid tumors, including HNSCC. Monalizumab has been shown to enhance its efficacy by promoting NK cell activity 43.

In the end, three genes causally associated with the risk of HNSCC were identified from the DEGs based on MR analysis. Among them, TKT serves as a protective factor for HNSCC, while LGALS2 and SLC11A1 act as risk factors.

## Conclusion

This paper extensively discusses the differences of ECT2 in terms of prognosis, immune microenvironment, drug sensitivity, etc. in HNSCC patients from the perspective of bioinformatics. Monocyte clusters were identified as high-scoring cell populations through ECT2 and genes interacting with it. The signaling pathways of communication between this cell population and other cell populations were explored. Through MR analysis, three DEGs in this cell population were identified as genes causally related to the risk of HNSCC.

## Supplementary Material

Supplementary figures.

## Figures and Tables

**Table 1:** Relationship between ECT2 expression and clinicopathological characteristics of TCGA-HNSC. cohort.

Characteristics	High expression of ECT2	Low expression of ECT2	p-value	Method
n	258	253		
**Age, n (%)**			0.417	Chisq test
<65	168 (32.9%)	156 (30.5%)		
≥65	90 (17.6%)	97 (19%)		
**Gender, n (%)**			0.004	Chisq test
MALE	205 (40.1%)	173 (33.9%)		
FEMALE	53 (10.4%)	80 (15.7%)		
**Grade, n (%)**			0.032	Chisq test
G1-G2	165 (33.7%)	194 (39.7%)		
G3-G4	74 (15.1%)	56 (11.5%)		
**Stage, n (%)**			0.179	Chisq test
Stage I-II	42 (9.6%)	55 (12.6%)		
Stage III-IV	173 (39.7%)	166 (38.1%)		
**T, n (%)**			0.225	Chisq test
T0-T2	84 (18.7%)	97 (21.6%)		
T3-T4	140 (31.2%)	128 (28.5%)		
**N, n (%)**			0.246	Chisq test
N0-N1	114 (27.7%)	125 (30.4%)		
N2-N3	92 (22.4%)	80 (19.5%)		

**Table 2:** Relationship between ECT2 expression and clinicopathological characteristics of self-selected cohort.

Characteristics	High expression of ECT2	Low expression of ECT2	p-value	Method
n	15	15		
**Age, n (%)**			0.715	Fisher test
<65	6 (20%)	8 (26.7%)		
≥65	9 (30%)	7 (23.3%)		
**Gender, n (%)**			0.143	Fisher test
MALE	10 (33.3%)	5 (16.7%)		
FEMALE	5 (16.7%)	10 (33.3%)		
**Grade, n (%)**			0.021	Fisher test
G2-3	13 (43.3%)	6 (20%)		
G1	2 (6.7%)	9 (30%)		
**Stage, n (%)**			0.427	Fisher test
Stage III-IV	12 (40%)	9 (30%)		
Stage I-II	3 (10%)	6 (20%)		
**T, n (%)**			0.710	Fisher test
T3-4	7 (23.3%)	5 (16.7%)		
T1-2	8 (26.7%)	10 (33.3%)		
**N, n (%)**			0.066	Fisher test
N1-2	11 (36.7%)	5 (16.7%)		
N0	4 (13.3%)	10 (33.3%)		
