# Supplementary material for: ECT2 promotes the occurrence and is a prognostic biomarker of head and neck squamous cell carcinoma
Source: J Cancer. 2024 Jun 3;15(13):4156–74. doi: 10.7150/jca.95515 (PMC11212090; doi:10.7150/jca.95515)
Supplement: Supplementary file 1 — Supplementary figures. [file jcav15p4156s1.pdf]

Supplementary Material

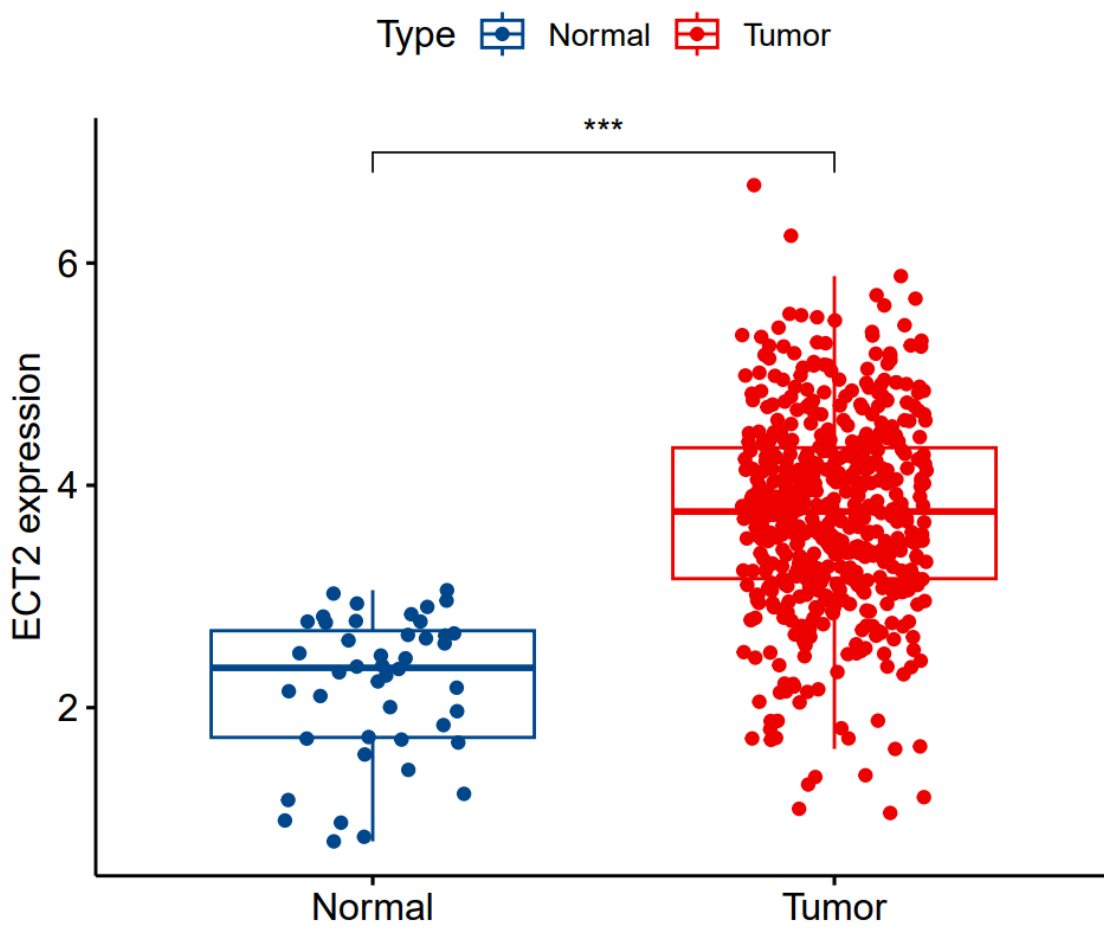

Figure S1 Boxplots of E2F1 expression in the control and JSNCC groups (* P<0.05, ** P<0.01, *** P<0.001)

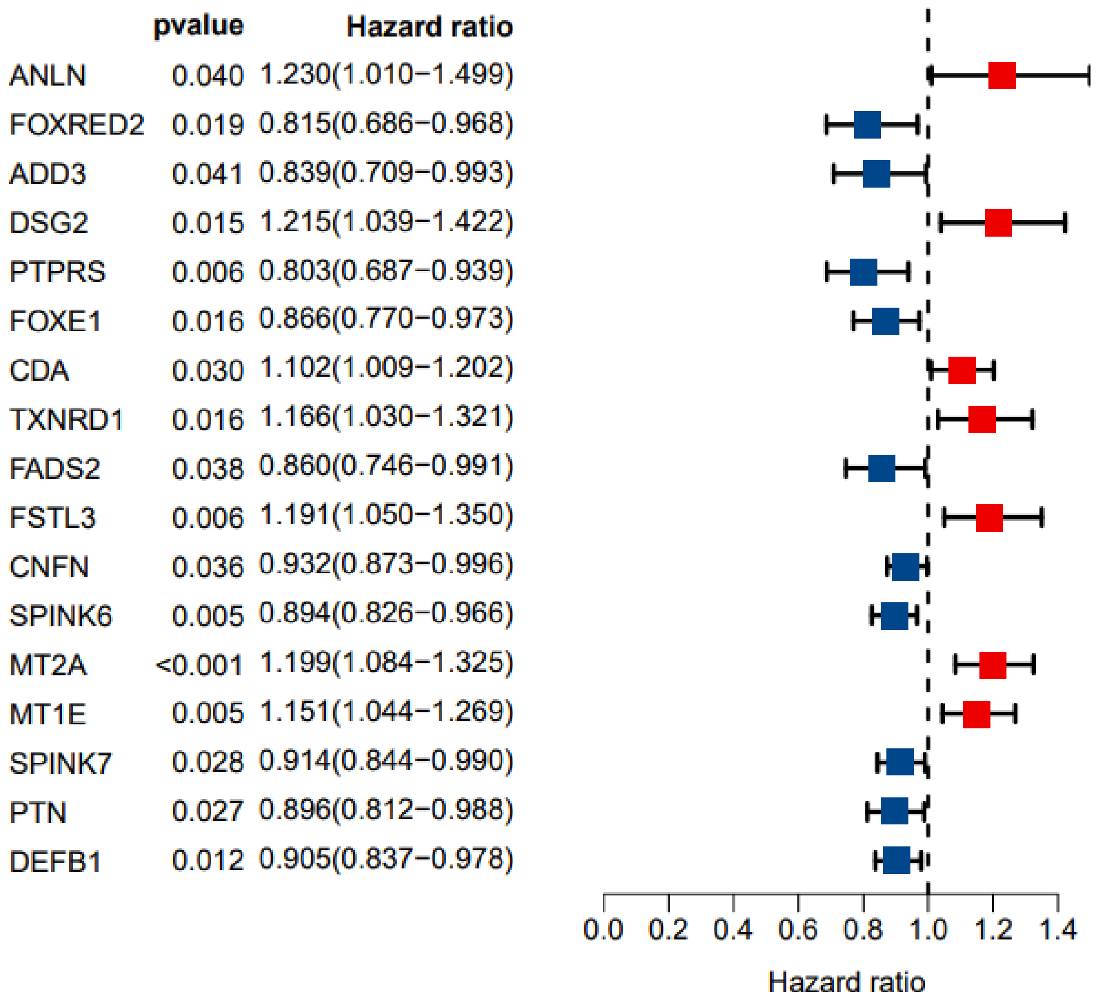

Figure S2 Forest plot obtained from single-factor Cox regression analysis

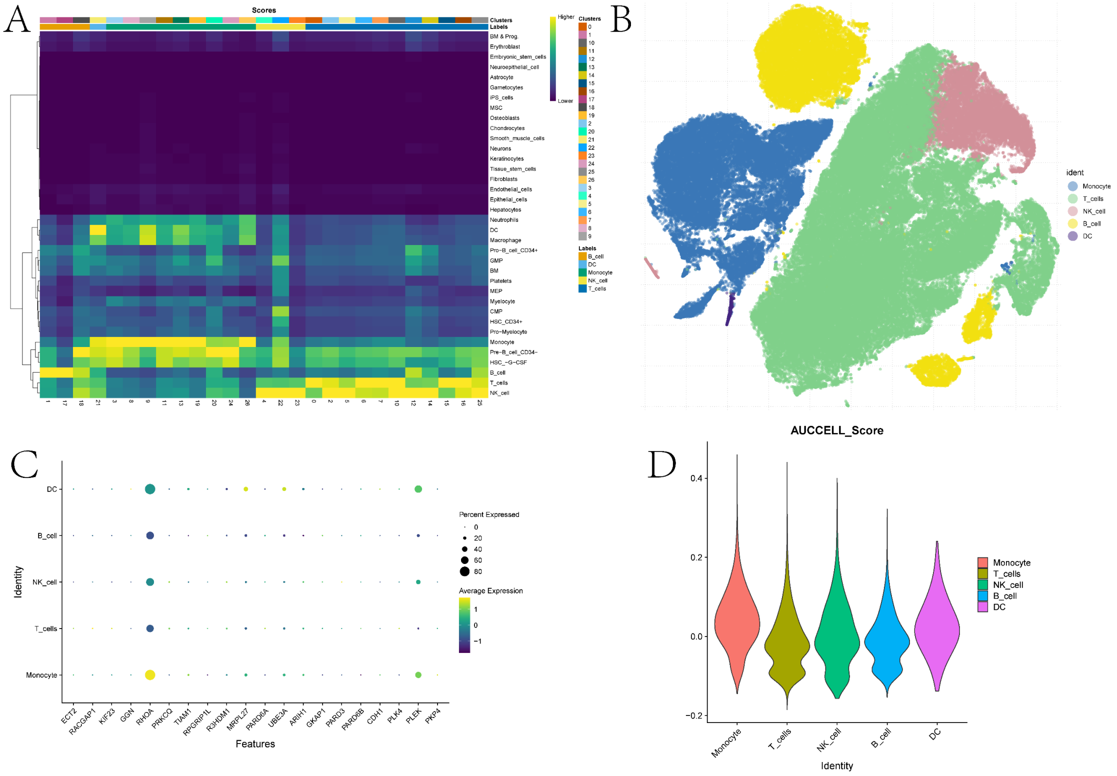

Figure S3 Cell type annotation and AUCell analysis results. A is a score heatmap predicting cell types based on the singleR package. B is a cell t-SNE scatter plot based on the final predictions from singleR. C is an expression heatmap of ECT2 and its interacting genes across different types of cell clusters. D is a violin plot of scores for different types of cells obtained based on the AUCell algorithm.

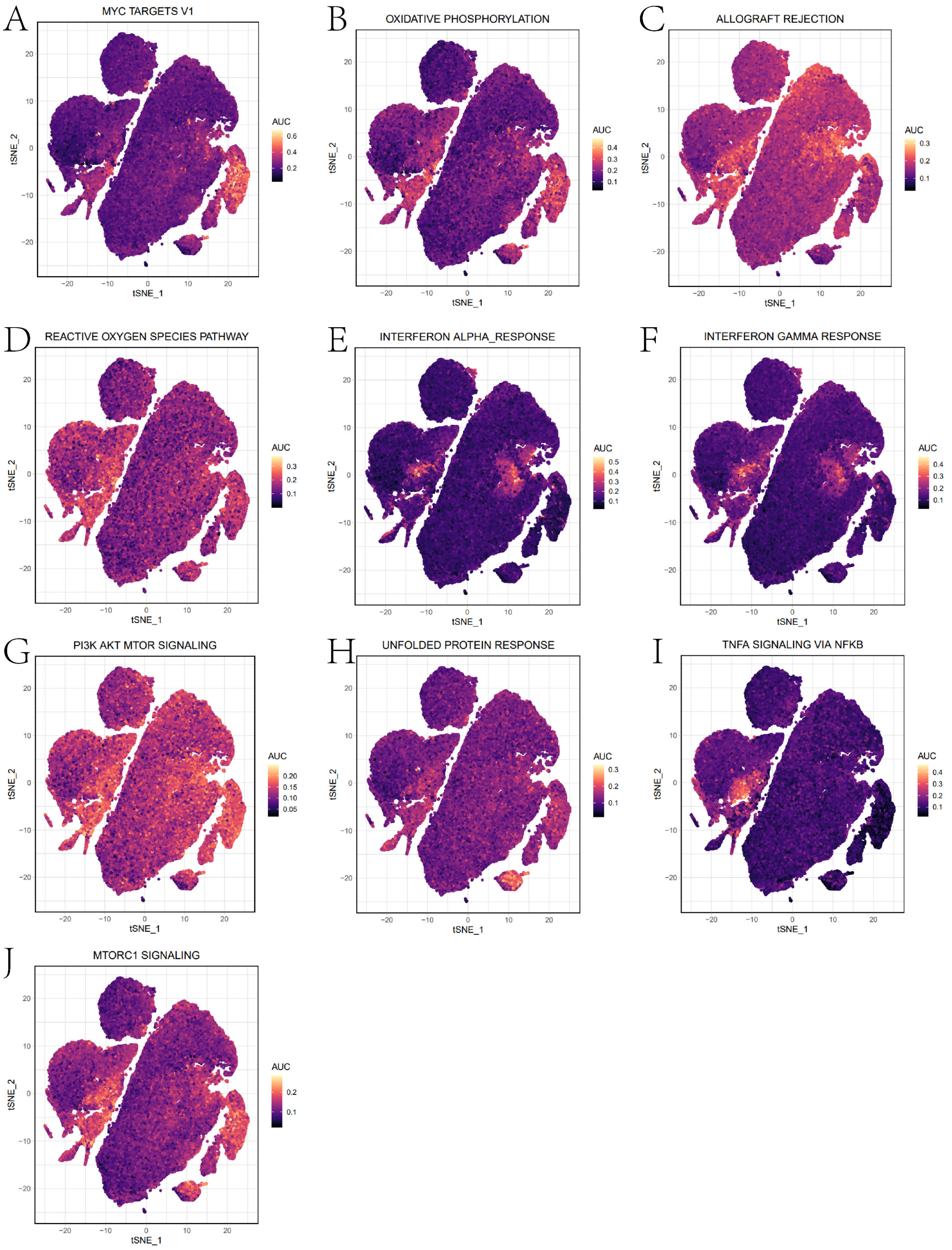

Figure S4 The top 10 pathways computed by the AUCell package

The weighted median model and IVW model of SLC11A1 support its causal relationship with head and neck cancer. The detailed results of the causal relationship analysis between SLC11A1 and the risk of head and neck cancer are shown in Figure S4. As shown in Figure S4A, the linear slopes of all five models are greater than 0, supporting a positive correlation between exposure and outcome. The forest plot in Figure S4B indicates that the IVW method supports that this gene is a risk factor for head and neck cancer. Funnel plot results indicate no directional horizontal pleiotropy

influencing IVW and weighted median estimates (Figure S4C). The forest plot in Figure S4D, derived from leave-one-out sensitivity analysis, indicates that the causal relationship is not driven by any single SNP with a strong influence.

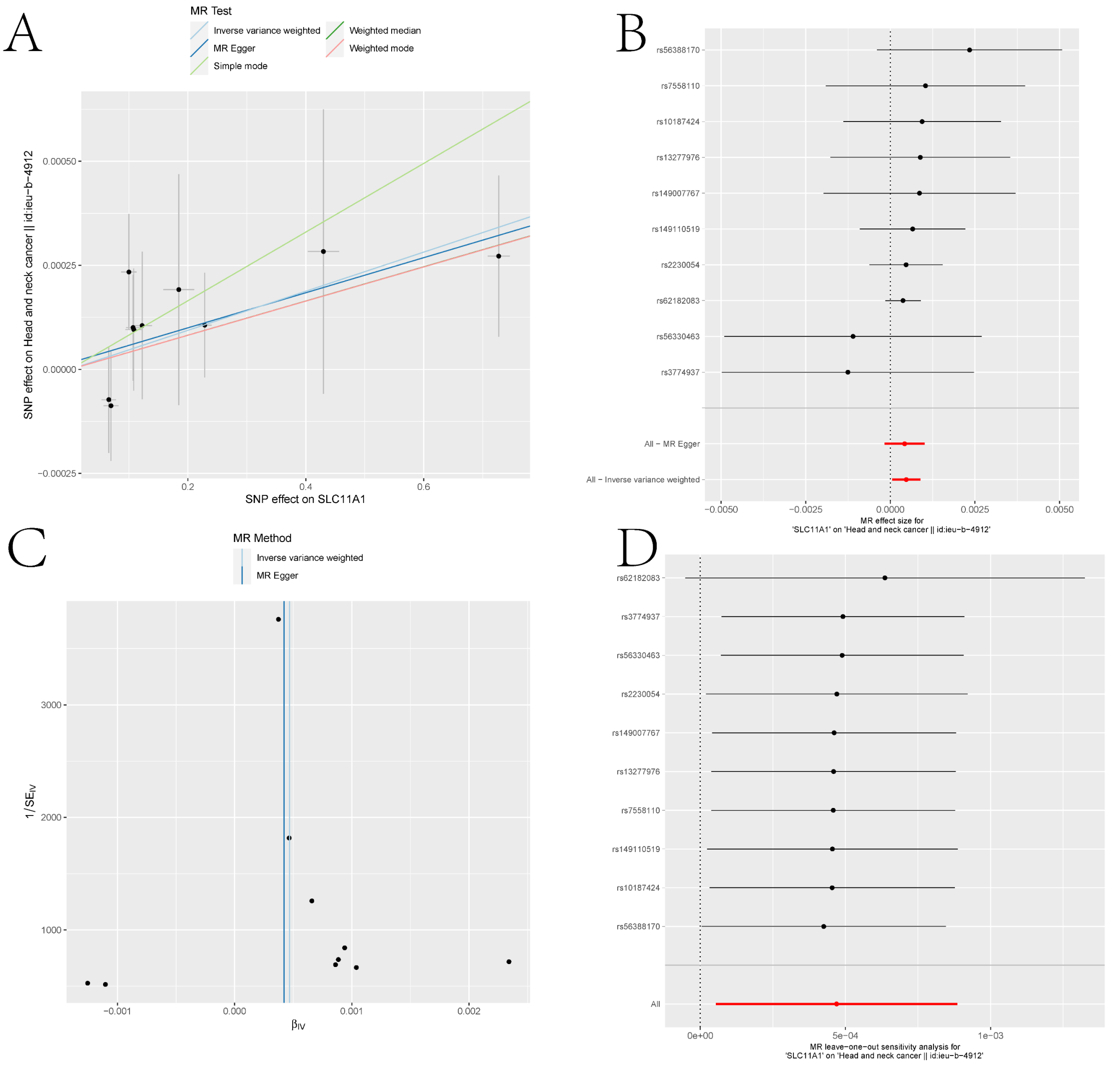

Figure S5 The MR analysis results of the causal relationship between SLC11A1 and the risk of head and neck cancer are as follows: A represents the impact of SNPs on head and neck cancer among five models. B represents the causal relationship of SLC11A1 on AD expressed by the Wald ratio. C and D are the results of sensitivity analysis.

The weighted median (TKT) and IVW models support their causal relationship with head and neck cancer. The detailed results of the causal relationship analysis between TKT and the risk of head and neck cancer are shown in Figure S5. From Figure S5A, it can be seen that the linear slopes of all five models are less than 0, supporting the negative correlation between exposure and outcome. The forest plot in Figure S5B indicates that the weighted median and IVW methods support that the gene is a protective factor for head and neck cancer. Funnel plot results indicate no directional horizontal pleiotropy affecting IVW and weighted median estimates (Figure S5C). The forest plot from leave-one-out sensitivity analysis in Figure S5D indicates that the causal relationship is not driven by any single SNP with strong influence.

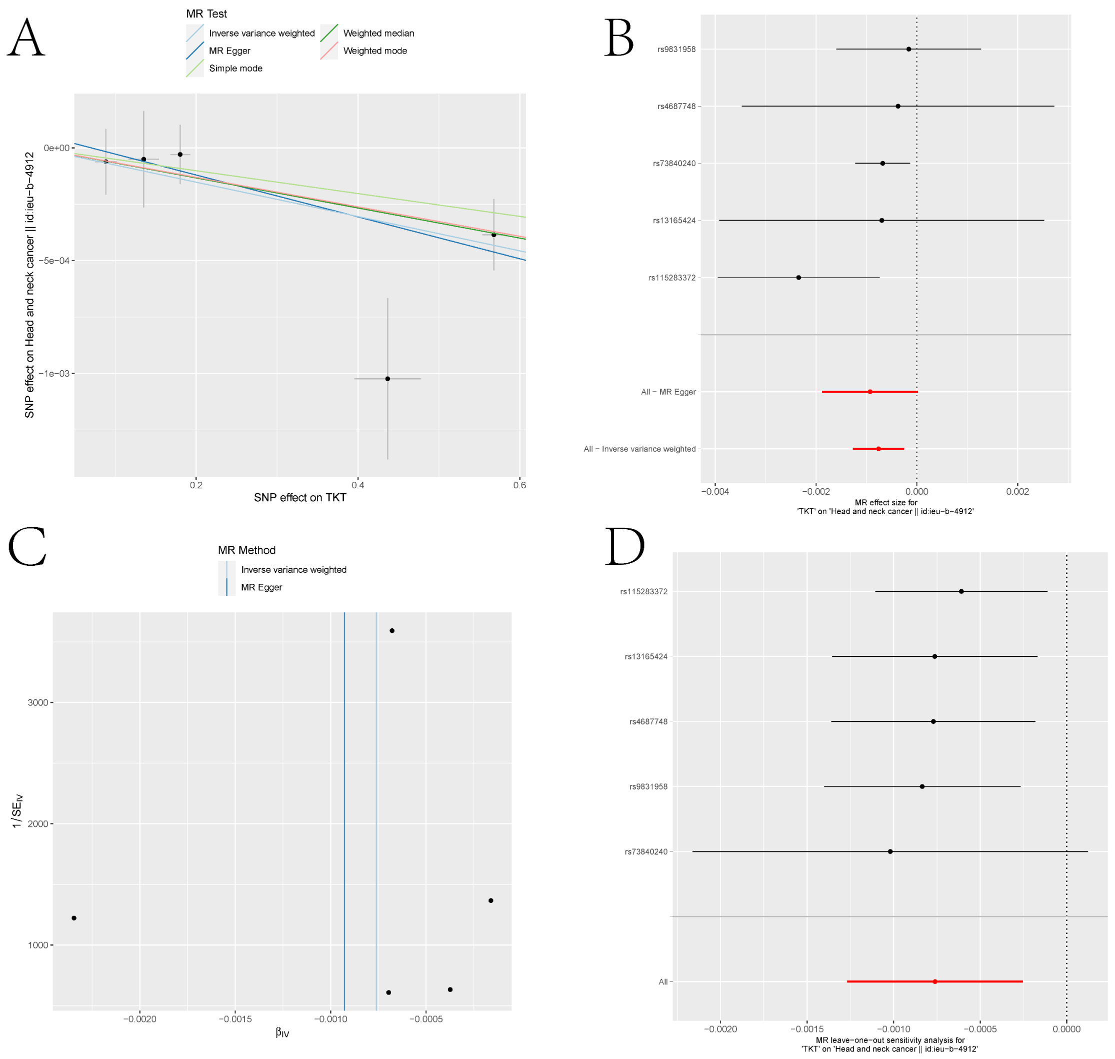

Figure S6 The MR analysis results of the causal relationship between TKT and head and neck cancer are as follows: A represents the impact of SNPs on head and neck cancer in five models. B represents the causal relationship of TKT to AD expressed by Wald ratio. C and D represent the results of sensitivity analysis.
